# 原发性肺黏液表皮样癌临床特征、*MAML2*基因重排和预后分析

**DOI:** 10.3779/j.issn.1009-3419.2025.101.10

**Published:** 2025-06-30

**Authors:** Jianrong BAI, Meng YAN, Lingchuan GUO, Zhe LEI, Weishuo LIU, Zigui ZOU, Jiao LI, Yushuang ZHENG

**Affiliations:** 215006 苏州，苏州大学附属第一医院病理科; Department of Pathology, The First Affiliated Hospital of Soochow University, Suzhou 215006, China

**Keywords:** 肺黏液表皮样癌, 分级, 预后, *MAML2*基因重排, Pulmonary mucoepidermoid carcinoma, Grade, Prognosis, *MAML2* gene rearrangement

## Abstract

**背景与目的** 原发性肺黏液表皮样癌（pulmonary mucoepidermoid carcinoma, PMEC）是起源于支气管黏液腺的罕见恶性肿瘤，占肺癌比例不足0.2%。其病理特征为黏液细胞、表皮样细胞及中间细胞混合构成，可分为低级别与高级别亚型，后者预后较差。本研究旨在探讨PMEC的临床病理特征及预后影响因素。**方法** 回顾性分析26例PMEC患者的临床资料、影像学表现、病理特征（含免疫组化及*MAML2*重排检测）及生存数据，结合文献进行总结。**结果** 26例患者中男性14例、女性12例，平均年龄55.6岁。吸烟者8例（30.8%），有症状者19例（73.1%）。中央型肿块19例（73.1%），周围型7例（26.9%）。计算机断层扫描（computed tomography, CT）均表现为低-中等密度团块/结节。病理分型：低级别19例，高级别7例。免疫组化示CK7、P40、P63、CK5/6阳性，Ki-67指数2%-70%。*MAML2*重排检出率52.4%（11/21）。临床分期：I期14例，II期8例，III期3例，IV期1例。治疗方式：根治手术13例，手术+辅助化疗11例，放化疗1例，保守治疗1例。中位随访57个月，6例（23.1%）死亡。预后分析显示：（1）高级别组生存率显著低于低级别组（*P*<0.05）；（2）淋巴结转移、晚期分期、Ki-67>20%及高级别与总生存期缩短有关联（*P*<0.05）；（3）淋巴结转移是独立不良预后因素（HR=12.73, 95%CI: 1.22-132.96）。**结论** PMEC具有独特的临床病理特征，约半数存在*MAML2*重排。淋巴结转移、晚期分期、高Ki-67指数及高级别是预后不良的影响因素，其中淋巴结转移为独立危险因素。

## Abstract (en)

**Background and objective** Primary pulmonary mucoepidermoid carcinoma (PMEC) is an exceedingly rare malignancy originating from bronchial submucosal glands, accounting for <0.2% of lung cancers. Histologically characterized by a triphasic composition of mucinous, epidermoid, and intermediate cells, PMEC is classified into low-grade (favorable prognosis) and high-grade (aggressive behavior) subtypes. This study aimed to investigate the clinicopathological characteristics and prognostic indicators of PMEC.**Methods** Clinicopathological, radiological, molecular, and survival data from 26 PMEC patients were retrospectively analyzed, including immunohistochemical profiles and *MAML2* rearrangement status, supplemented by literature review.**Results** The cohort comprised 14 males and 12 females (mean age: 55.6 years). Eight patients (30.8%) were smokers, and 19 (73.1%) presented with symptoms. Central tumors predominated (*n*=19, 73.1%) versus peripheral lesions (*n*=7, 26.9%). Computed tomography (CT) imaging consistently revealed hypo-to-isodense masses/nodules. Pathologically, 19 cases were low-grade and 7 high-grade. Immunohistochemically, the tumor cells were positive for CK7, P40, P63 and CK5/6, and the Ki-67 index ranged from 2% to 70%. *MAML2* rearrangement was detected in 52.4% (11/21) of tested cases. Clinical staging distribution: stage I (*n*=14), stage II (*n*=8), stage III (*n*=3), stage IV (*n*=1). Treatment modalities: radical surgery alone (*n*=13), surgery with adjuvant chemotherapy (*n*=11), chemoradiotherapy (*n*=1), and conservative management (*n*=1). With a median follow-up of 57 months, 6 patients (23.1%) died. Prognostic analysis demonstrated: (1) Significantly inferior survival in high-grade versus low-grade groups (*P*<0.05); (2) Lymph node metastasis, advanced stage, Ki-67>20%, and high-grade histology significantly correlated with reduced overall survival (*P*<0.05); (3) Lymph node metastasis constituted an independent poor prognostic factor (HR=12.73, 95%CI: 1.22-132.96).**Conclusions** PMEC exhibits distinct clinicopathological features, with *MAML2* rearrangement present in approximately half of cases. Lymph node metastasis, advanced stage, high Ki-67 proliferation index, and high-grade histology are key determinants of poor prognosis, with lymph node metastasis serving as an independent risk factor.

原发性肺涎腺型肿瘤（primary pulmonary salivary gland-type tumor, PSGT）是一种起源于支气管黏膜下腺体的罕见肿瘤，其仅占所有原发性肺癌比例不到1%^[1]^。PSGT中较常见的亚型为肺黏液表皮样癌（pulmonary mucoepidermoid carcinoma, PMEC）和肺腺样囊性癌（pulmonary adenoid cystic carcinoma, PACC），两者发病率均小于0.2%且前者占比最大^[2,3]^。PMEC的特征性病理表现是包含表皮样细胞、中间细胞和黏液分泌细胞三种成分。通常根据组织学表现（包括有丝分裂频率、细胞异型性等）可将PMEC分为低级别或高级别，其中低级别患者预后较好^[3]^。值得注意的是，66%-100%的PMEC中存在特征性的t（11; 19）易位，导致*MECT1-MAML2*（mucoepidermoid carcinoma translocated 1 mastermind-like 2）融合基因形成^[3]^。目前，手术切除仍然是PMEC的主要治疗方法，而化疗或放疗的疗效证据有限。PMEC具有与其他常见肺部肿瘤不同的临床、病理、分子和生物学特征，加之其罕见性，容易造成漏诊或误诊。因此，其诊断需要结合临床、实验室、影像资料排除转移性疾病，并通过组织病理学检查和必要的分子检测来进行诊断与鉴别，以指导临床管理与治疗。本研究回顾性分析本中心收治的26例PMEC患者资料，并深入探讨临床病理特征与预后的关系，以期深化对该疾病的认识并为精准诊疗提供依据。

## 1 资料与方法

### 1.1 病例选择

收集苏州大学附属第一医院2009年6月至2023年12月收治的经病理确诊的PMEC患者26例，诊断标准参照世界卫生组织（World Health Organization, WHO）（2021）分类^[1]^。整理患者的临床特征、病理学特征、治疗策略和随访资料并进行回顾性分析，本研究获苏州大学附属第一医院伦理委员会批准[批准号：（2025）伦审批第063号]。

### 1.2 免疫组织化学及特殊染色

患者组织标本经10%中性缓冲福尔马林液固定、常规脱水、石蜡包埋并以4 μm厚度进行切片。组织切片行苏木精-伊红（hematoxylin-eosin, HE）染色、免疫组织化学EnVision法染色和过碘酸雪夫（periodic acid-schiff stain, PAS）染色。所用一抗包括：细胞角蛋白7（cytokeratin 7, CK7）、甲状腺转录因子1（thyroid transcription factor 1, TTF-1）、P63、CK5/6、CD117、Calponin和Ki-67（基因科技公司）；Napsin A和P40（中杉金桥公司），S100、SMA和程序性死亡配体1（programmed death-ligand 1, PD-L1）（克隆号22C3，DAKO公司）。所有试剂相关操作步骤均严格按照说明书进行。

### 1.3 荧光原位杂交（fluorescence *in situ* hybridization, FISH）检测

采用针对*MAML2*基因位点的断裂探针（广州安必平医药科技股份有限公司）进行FISH检测。检测步骤均严格按试剂说明书操作。

### 1.4 诊断判读

所有病例的原始HE切片及诊断由2位高年资病理医师独立复核，包括组织学亚型确认和分级评估，对于复核结果差异较大的病例，邀请第3位高年资病理医师复片以达成共识。在免疫组化切片中，通过显微镜观察多个代表性视野，计数肿瘤细胞总数及Ki-67阳性（细胞核染色）细胞数，计算阳性细胞占比（Ki-67阳性细胞数/肿瘤细胞总数×100%），取多个视野平均值作为最终Ki-67指数。FISH检测后观察肿瘤细胞核检测结果并进行计数和比值计算，阴性细胞为2个融合信号，当肿瘤细胞呈现红、绿信号分离≥2个信号点直径之和，判读为分离信号；计数至少100个形态学明确的肿瘤细胞核，若显示分离信号的细胞比例>15%则判读为FISH阳性。

### 1.5 随访

通过电话随访及查阅电子病历系统获取患者治疗信息及生存状态。随访时间定义为确诊日期至死亡日期或末次随访时间（2024年11月30日）。随访时间11-185个月，中位随访时间为57个月。

### 1.6 统计学方法

通过SPSS 软件（26.0 版）进行数据分析。采用*Kaplan-Meier*法评估不同组别生存情况，组间生存差异通过对数秩检验（*Log-rank* test）进行统计比较。使用单因素和多因素*Cox*比例风险回归模型估计影响生存的潜在因素，其中模型开展多因素回归的自变量筛选条件为：经多重共线性检验方差膨胀因子（variance inflation factor, VIF）值<10并满足单因素分析*P*<0.2。以*P*<0.05为差异具有统计学意义。

## 2 结果

### 2.1 临床特征

26例PMEC中男性14例，女性12例，年龄、28-75岁（平均年龄55.6岁）。30.8%（8/26）的患者有吸烟史。首次就诊时无症状（体检发现）者7例（26.9%），有症状者19例，常见症状包括咳嗽、咳痰、胸痛和发热。首次获得诊断性组织标本的方法为：外科手术切除标本18例（69.2%），支气管镜活检标本8例（30.8%）。

### 2.2 影像学特征

影像学上，肿瘤位于左肺17例，右肺9例。根据肿瘤主体位置与支气管关系分型：中央型19例（位于主/叶/段支气管），周围型7例（位于肺叶实质）。肿块最大直径范围1.5-7.0 cm（平均直径3.3 cm），其中最大径≤3 cm者16例，>3 cm者10例。计算机断层扫描（computed tomography, CT）表现（图1）为密度不均的低-中等密度肺部团片状影或结节影，多数为中央型的气管-支气管腔内边界尚清的软组织结节或肿块，其中7例支气管远端出现阻塞性肺炎征象，部分病例可出现肿块内坏死、空洞，肺门及纵隔淋巴结转移，增强扫描后病灶多呈轻-中度不均匀强化。

**图1:**
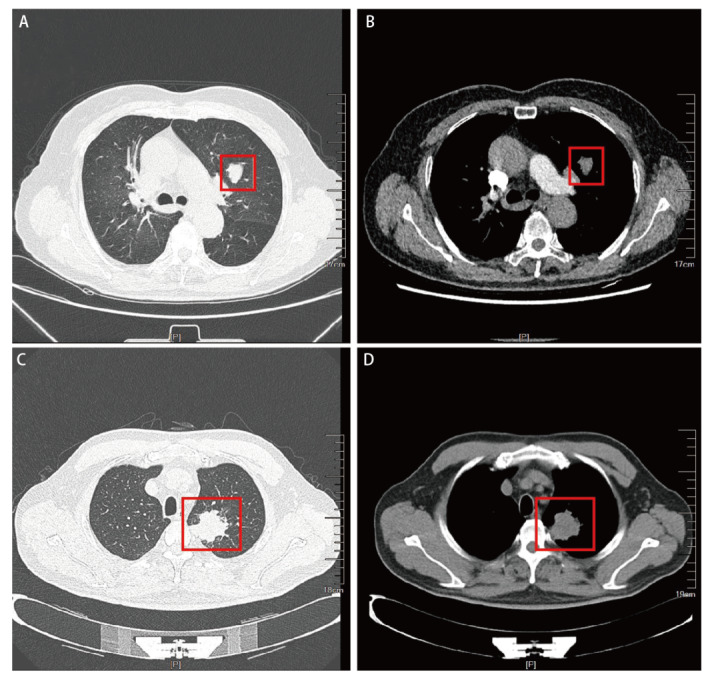
影像学表现。 A-B：病例5，女性，66岁，CT平扫（图1A）显示左肺上叶见一团块状软组织密度影（22 mm×16 mm），增强CT（图1B）可见强化，术后经病理评估为低级别PMEC；C-D：病例9，男性，58岁，CT平扫（图1C）显示左肺上叶尖后段见一团块状软组织密度影（41 mm×37 mm），其内可见小片稍低密度灶，境界尚清，边缘不规则，呈分叶状，可见毛刺征，增强CT（图1D）可见不均匀明显强化，术后经病理评估为高级别PMEC。

### 2.3 病理学特征

PMEC肿瘤组织由黏液细胞、表皮样细胞和中间细胞组成，常形成囊腔，间质为数量不等的纤维结缔组织分隔。黏液细胞呈柱状、立方状或透明细胞样；表皮样细胞呈多边形，缺乏明显的角化和细胞间桥；中间细胞椭圆或多边形，胞浆嗜酸性或嗜双色。根据不同细胞的丰度、坏死的存在、细胞核异型性和核分裂象，PMEC分为高级别和低级别两种组织学分级，高级别者较少见^[4]^。本组病例（图2A-2B）中：（1）高级别：7例（7/26），以实性结构为主，由分化较差的中间细胞和表皮样细胞构成，黏液细胞较少（<10%）。有明显的核分裂（>4个/10HPF）和细胞异型性，坏死较常见。（2）低级别：19例（19/26），由腺体、小管、囊肿和实性区集合组成，大部分为腺管结构。三种细胞成分均可见，分化良好，以黏液细胞（≥10%）和表皮样细胞常见，细胞异型性轻微，无或极少有核分裂和坏死。所有PMEC病例中，3例患者手术标本支气管切缘镜下见肿瘤累及，6例患者存在区域淋巴结转移。此外，1例患者肺内同时存在PMEC（低级别）和一处独立的中分化腺癌（考虑为双原发肿瘤）。根据第九版国际抗癌联盟肿瘤原发灶-淋巴结-转移（tumor-node-metastasis, TNM）分期：I期14例，II期8例，III期3例，IV期1例。肿瘤细胞阳性表达CK7、P40、P63和CK5/6，而TTF-1、Napsin A、S100、Calponin、SMA不表达。Ki-67增殖指数差异较大，范围为2%-70%，Ki-67指数≤20%者19例（73.1%），指数>20%者7例（26.9%）。低级别组平均Ki-67指数为14.7%，显著低于高级别组的44.6%。黏液细胞AB/PAS染色呈阳性。5例患者接受了PD-L1（22C3）检测，结果显示除1例因肿瘤细胞阳性比例评分（tumor proportion score, TPS）<1%而判读为阴性外，其他4例判读为阳性（TPS≥1%）。值得注意的是，PD-L1阳性的4例中，3例为高级别PMEC，1例为低级别PMEC。典型免疫组化和特殊染色结果示例见图2C-2H。

**图2:**
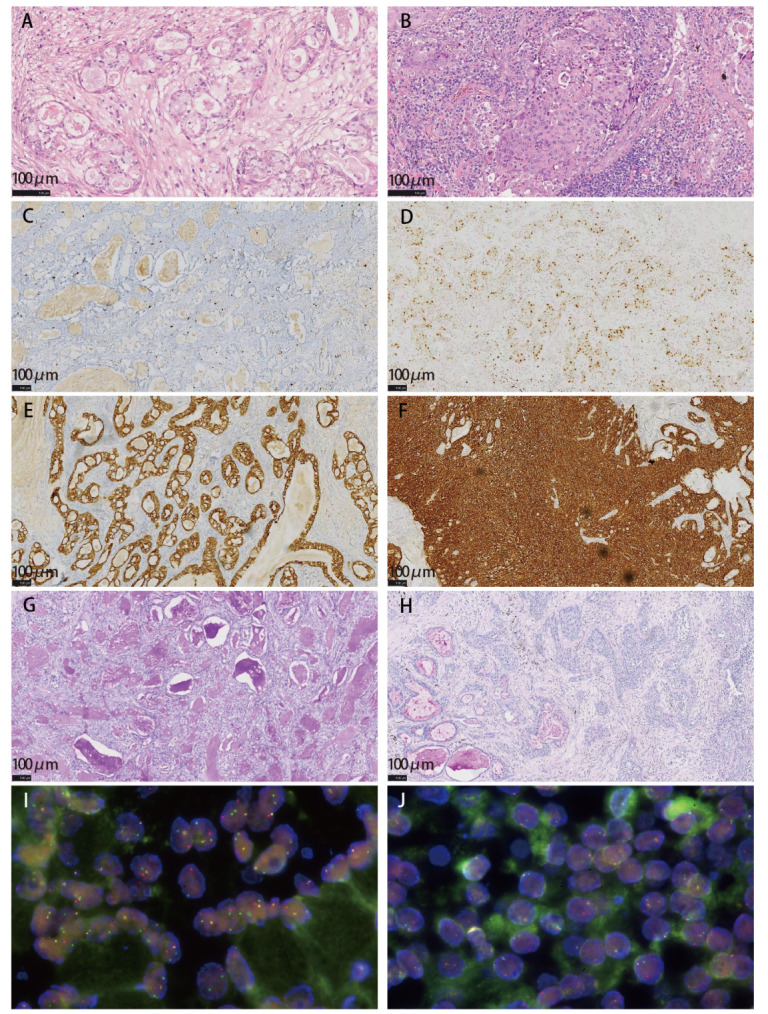
病理学典型表现。 A：低级别PMEC的HE：可见分化良好的黏液细胞形成腺样腔隙或囊腔，细胞异型性小；B：高级别PMEC的HE：可见分化较差的表皮样细胞和中间细胞呈片状、岛状分布，细胞异型性明显，核仁显著，可见核分裂；C：低级别PMEC的Ki-67（约5%）；D：高级别PMEC的Ki-67（约30%）；E：低级别PMEC的CK7阳性，标记肿瘤组织腺管样区域；F：高级别PMEC的CK7阳性，标记肿瘤组织弥漫片状区域；G：低级别PMEC的PAS染色，标记大量黏液细胞；H：高级别PMEC的PAS染色，标记小灶区域黏液细胞；I：低级别PMEC FISH阳性病例示意图（×1000）；J：高级别PMEC FISH阳性病例示意图（×1000）。

### 2.4 分子检测结果

21例顺利获取MAML2 FISH检测结果。分子遗传学上，本研究PMEC患者*MAML2*基因重排检出率为52.4%（11/21），其中低级别组（n=15）的检出率为66.7%（10/15），显著高于高级别组（n=6）的16.7%（1/6）。典型MAML2 FISH检测结果见图2I-2J。

### 2.5 治疗与预后分析

本研究选择总生存期（overall survival, OS）作为主要终点，基于PMEC的低度恶性潜能和相对较低的复发风险^[5]^，并结合本队列样本量情况。全部26例PMEC患者中，13例患者接受了单纯根治性手术治疗（其中2例死亡），11例接受了根治性手术+术后辅助化疗（其中3例死亡），1例经活检后仅接受了放疗+化疗（死亡），1例经活检后仅接受保守观察（存活）。预后上，随访期间所有病例中：6例死亡；6例发生区域淋巴结转移（其中4例死亡）；3例手术标本支气管切缘阳性，其中1例死亡（该患者同时伴有淋巴结转移，OS为49个月）。PMEC低级别组（n=19）死亡2例（10.5%），高级别组（n=7）死亡4例（57.1%）。*Kaplan-Meier*生存分析（图3）显示，高级别PMEC患者的总生存率显著低于低级别患者（*P*=0.010）。单因素*Cox*分析（表1）显示淋巴结转移、晚期分期、Ki-67指数>20%及高级别与较差的OS有关联（*P*<0.05）。进一步的多因素*Cox*回归分析（表1）确认淋巴结转移是影响OS的独立危险因素（HR=12.73, 95%CI: 1.22-132.96）。

**图3:**
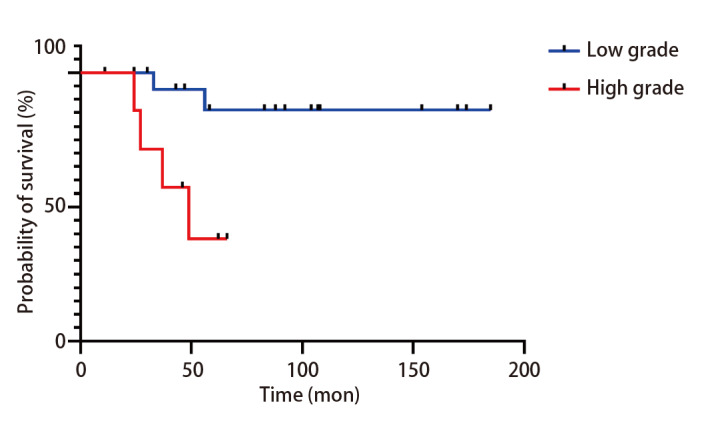
低级别和高级别组的*Kaplan-Meier*总生存曲线

**表 1:** PMEC单因素和多因素*Cox*比例风险模型结果

Variables	*n*	Univariate analysis						Multivariate analysis				
		*β*	S.E.	*Z*	*P*	HR (95%CI)		*β*	S.E.	*Z*	*P*	HR (95%CI)
Gender												
Female	12					1.00 (Reference)						1.00 (Reference)
Male	14	1.68	1.10	1.53	0.125	5.39 (0.63-46.35)		0.99	1.13	0.88	0.380	2.69 (0.29-24.58)
Smoking												
No	18					1.00 (Reference)						
Yes	8	0.94	0.82	1.15	0.250	2.57 (0.51-12.79)						
Symptom												
No	7					1.00 (Reference)						
Yes	19	-0.59	0.87	-0.68	0.495	0.55 (0.10-3.03)						
Location												
Peripheral	7					1.00 (Reference)						
Central	19	-0.90	0.87	-1.03	0.304	0.41 (0.07-2.26)						
Tumor size (cm)												
≤3	16					1.00 (Reference)						
>3	10	0.57	0.82	0.70	0.483	1.77 (0.36-8.81)						
Lymph node metastasis												
No	20					1.00 (Reference)						1.00 (Reference)
Yes	6	3.19	1.15	2.78	0.005	24.20 (2.56-229.20)		2.54	1.20	2.13	0.034	12.73 (1.22-132.96)
T-stage												
1	14					1.00 (Reference)						
2	7	-0.35	1.16	-0.30	0.760	0.70 (0.07-6.77)						
3	5	0.73	0.92	0.79	0.429	2.06 (0.34-12.47)						
Clinical stage												
1	14					1.00 (Reference)						
2	8	-0.24	1.23	-0.20	0.845	0.79 (0.07-8.70)						
3	3	3.26	1.33	2.46	0.014	26.10 (1.94-351.25)						
4	1	26.59	60,129.83	0.00	>0.999	352,832,966,979.32 (0.00-Inf)						
Ki-67 index												
≤20%	19					1.00 (Reference)						
>20%	7	1.94	0.87	2.22	0.026	6.98 (1.26-38.71)						
Age (yr)												
≤55	11					1.00 (Reference)						
>55	15	21.22	16,445.47	0.00	0.999	1,646,429,906.28 (0.00-Inf)						
Grade												
Low	19					1.00 (Reference)						1.00 (Reference)
High	7	1.94	0.87	2.22	0.026	6.98 (1.26-38.71)		0.79	0.96	0.83	0.409	2.20 (0.34-14.38)
Positive surgical margin												
No	21					1.00 (Reference)						
Yes	3	0.55	1.12	0.48	0.628	1.72 (0.19-15.63)						
*MAML2* gene rearrangement												
No	10					1.00 (Reference)						
Yes	11	-0.66	0.91	-0.72	0.471	0.52 (0.09-3.10)						

Clinical stage and Ki-67, which met *P*<0.2 in univariate analysis, were removed from the multivariate cox regression model analysis because they did not meet VIF value<10 to avoid parameter estimation bias.

PMEC: pulmonary mucoepidermoid carcinoma; HR: hazard ratio; CI: confidence interval.

## 3 讨论

PMEC是一种极为罕见的肺部肿瘤，受限于其低发病率，现有文献多基于个案报道或小样本研究。本研究回顾性分析了26例经病理确诊的PMEC患者，进一步丰富了对该疾病的认识。本研究显示PMEC患者年龄跨度大，好发于中老年人群，且男女发病率相近。PMEC通常起病隐匿，其临床症状缺乏特异性^[6]^。本研究观察到肿瘤发生部位以中央型为主（73.1%），这与部分文献^[7]^报道的周围型更常见存在差异，可能与研究样本局限性和多样性有关。患者早期常无明显症状，后期因瘤体占位可出现刺激性咳嗽、咳痰、发热、胸闷、胸痛、呼吸困难等症状^[8]^。本研究中较高的无症状患者比例（26.9%）凸显了影像学筛查在早期发现中的重要性。迄今为止，尚未有充足的文献证据证明吸烟是PMEC明确的致病危险因素^[9]^。

临床上，及时和完善的辅助检查对最终正确诊断至关重要。PMEC通常生长隐匿且缓慢，但具有局部浸润性，计算机断层扫描（computed tomography, CT）上病灶可表现为密度不均的腔内、腔内外、周围型分叶状或结节状肿块，有时伴有远端阻塞性改变，少数肿瘤可延伸到纵隔及其他部位^[10]^。既往研究^[11]^发现相比PACC，PMEC更易发生坏死、囊性变、钙化及空洞形成，推测可能与两者在组织结构、细胞成分及血管分布等方面的差异有关，更多影像特征有待更大样本的研究来系统总结和验证。支气管镜下多表现为息肉状或结节状腔内新生物，表面常无完整包膜，并可见管壁局部浸润性增厚。鉴于部分PMEC临床症状缺乏特异性，故对原因不明、治疗效果欠佳的呼吸道症状者应积极考虑行支气管镜检查配合病理活检，以避免漏诊。

组织病理学检查是确诊PMEC的“金标准”。大体上，肿瘤多表现为支气管腔内灰白色、质实、不规则结节状肿块。镜下，特征性的3种细胞成分混合存在，形成实性或腺囊性结构，间质可见纤维分隔。本研究26例PMEC中，低级别组占73.1%（19例），高级别组占26.9%（7例），这种低级别占主导的比例特征符合临床实际并与其他研究^[12,13]^报道类似。PMEC主要鉴别诊断是腺鳞癌、鳞状细胞癌、玻璃样变透明细胞癌、腺泡细胞癌、具有胞浆内黏蛋白的实体腺癌、反应性支气管腺体等。本研究中肿瘤细胞免疫组化表达特征与相关研究^[12]^一致，部分免疫组化有助于鉴别诊断，例如：TTF-1/Napsin A阴性有助于排除肺腺癌或腺鳞癌成分；有研究^[14]^提示P63在PMEC中可能比P40更敏感且表达更强。值得注意的是，PMEC无法单纯依赖免疫标记进行鉴别，需要结合镜下组织学特征和分子检查结果综合判断。转移性MEC肿瘤形态、免疫表型和黏液染色等特征与原发于涎腺者较一致，而肺是最常见的血行性转移部位，因此PMEC诊断必须排除转移可能。本研究证实高级别组平均Ki-67增殖指数显著高于低级别组，且在生存分析中发现Ki-67指数>20%与患者不良预后密切相关，这也为临床评估预后提供了一个潜在的实用阈值。因此，CK7、TTF-1、Napsin A、P63、P40、Ki-67、PAS染色的联合检测或可成为PMEC辅助诊断和分级的重要指标组合。

典型的组织学形态结合免疫表型一般可以做出PMEC诊断，必要时可结合分子检测协助诊断。分子遗传学上，PMEC的特征性改变是t（11;19）（q21;p13）染色体易位导致的*MECT1-MAML2*融合基因形成。该融合事件在肺的低分化腺癌或腺鳞癌中不存在，因此*MAML2*重排检测对PMEC诊断具有高度特异性。*MAML2*重排存在于40%-90%的头颈MEC中和50%-100% 的PMEC中^[15]^，我们的研究数据中该比例为52.4%。机制层面，研究^[16]^表明*MECT1-MAML2*融合产物通过干扰正常Notch信号转导机制，独立激活Notch靶基因和多重cAMP/CREB的转录，从而阻断Notch和环磷腺苷效应元件结合蛋白（cAMP-response element binding protein, CREB）信号转导通路，诱导肿瘤形成。本研究中*MAML2*重排存在显著的病理分级相关性：低级别组检出率为66.7%，而高级别组仅为16.7%，但未发现明确预后相关性。既往研究^[17]^发现*MAML2*重排在低级别组中更常见，并常提示更良好的预后，而在高级别组中似乎不太常见，如果存在，其预后也可能优于*MAML2*阴性者。除了*MECT1-MAML2*易位之外，PMEC的其他分子研究非常有限且结果存在不一致。例如，一项研究^[18]^报道了约25%（5/20）的PMEC存在人表皮生长因子受体（epidermal growth factor receptor, EGFR）基因21外显子L861Q突变，而另一项纳入26例PMEC患者的研究^[12]^在包括EGFR、KRAS和BRAF等在内的多种常见驱动基因中均未发现突变。这些差异可能源于研究人群、检测方法或样本量的不同，同时也凸显了该领域研究的挑战性。因此，未来需要进一步的调查来系统探索其他潜在分子标志物在PMEC中的发生频率、临床意义及治疗价值。

本研究通过生存曲线量化了PMEC组织学分级与预后的关联，证实高级别PMEC患者总生存显著劣于低级别组，这与该肿瘤的生物学行为特征一致。Li等^[13]^研究发现IV期、分化程度差（即高级别）和淋巴结转移是PMEC的危险因素，且高级别是独立预后因子。Huo等^[12]^研究指出年龄≥50岁、支气管周围生长模式、肿瘤大小≥3 cm和Ki-67标记指数≥10%都是不良预后因素。本研究的Cox回归分析进一步证实：淋巴结转移、分期较晚、Ki-67增殖指数>20%和高级别组织学对患者OS有显著影响，且淋巴结转移是影响不良预后的独立危险因素。不同病理分级在预后分层中显示出重要意义，虽然*MAML2*基因重排在低级别PMEC具有高发生率，但在本研究中*MAML2*基因重排与预后的关联未达统计学显著性，而其他研究中该结果也不完全一致^[12,19]^，因此未来需要进一步扩大样本研究其在PMEC中的生物学意义。一项基于监测、流行病学和结果数据库（Surveillance, Epidemiology, and End Results, SEER）的大样本（n=585）研究^[20]^结果显示年龄>60岁、双侧肿瘤、肿瘤大小>3 cm、病理分化差、淋巴结转移和远处转移是PMEC患者生存差的独立预后因素。病理分级的高低在评估PMEC患者生存情况中发挥着关键作用，与低级别PMEC相比，高级别PMEC更容易复发和转移^[7]^。临床分期也有助于对患者进行预后判断和分层管理，经统计，III-IV期PMEC的5年疾病特异性生存率（31%）远低于I-II期（91%）^[20]^，凸显了早期诊断和治疗的重要性。

PMEC的治疗多以外科手术治疗为主，此类肿瘤一般进展缓慢，病程较长，转移出现晚，预后较好。本组数据及其他研究^[20,21]^均显示，接受根治性手术的患者生存结局优于非手术治疗（如放化疗）。手术应力求达到R0切除（镜下切缘阴性），切缘阳性（R1/R2）的患者后期容易出现复发/转移。对于晚期（III-IV期）、切缘阳性或伴有淋巴结转移的患者，术后辅助治疗（放疗±化疗）常被采用，尽管其确切效益仍需更高级别证据支持，因为PMEC对传统放化疗相对不敏感。免疫检查点抑制剂（immune checkpoint inhibitors, ICIs）在PMEC中的潜在价值值得关注。程序性死亡受体1（programmed cell death 1, PD-1）在活化的T细胞上表达，与配体PD-L1和PD-L2结合后抑制效应T细胞功能。有研究^[22]^探索了恶性涎腺肿瘤中的PD-L1和PD-L2表达，发现MEC患者PD-L1的肿瘤细胞评分和免疫细胞评分均显著高于其他涎腺肿瘤患者。在PMEC中，Gyulai等^[23]^观察到2例（7.7%）PD-L1在肿瘤细胞上表达，9例（34.6%）PD-L1在免疫细胞上表达。本组有限的PD-L1检测（n=5）显示4例阳性（TPS≥1%），其中3例病理分级属于高级别（2例生存，1例死亡），1例属于低级别（生存）。本研究中患者均未接受ICIs治疗，因此PD-L1表达状态与PMEC的肿瘤微环境、免疫疗效和生存期是否存在关联有待进一步考证和探索。此外，一些新的治疗方式也显示出一些前景，例如经支气管镜介入治疗、碳离子（C^12^）强子辐照和酪氨酸激酶抑制剂靶向疗法展现出初步潜力^[3,24]^，亟待更多临床研究验证。

作为一项回顾性研究，这些数据的收集和审查仍然存在一些局限性，例如，由于较低的发病率，本研究纳入的样本数量相对较少，相关检测（如PD-L1、*MAML2*）覆盖率有限。此外，患者人群特征（地域、种族）、数据共线性、随访时间跨度、患者本身医疗条件及治疗方式等都可能影响结论的可靠性和准确性，导致结果偏倚，我们计划通过未来多中心、大样本的高质量前瞻性研究以克服上述局限并深入探索和评估。

综上所述，本研究通过整合分析26例PMEC患者的相关资料并结合文献复习，系统阐述了这一罕见肿瘤的独特临床病理特征。本研究证实了PMEC患者的年龄跨度较大而男女比例相近；肿瘤部位以中央型常见；组织学分级为低级别者占比较高；约半数存在*MAML2*基因重排，且其在低级别组中具有较高发生率；淋巴结转移、分期较晚、高Ki-67指数及高级别是PMEC预后不良的影响因素，其中淋巴结转移是独立危险因素。这些发现有助于深化临床对PMEC的认识，为早期诊断、精准病理评估及个体化治疗决策提供依据，最终有望改善患者生存结局。
